# Microbial spore genetic marker technology, a potential technology for traditional Chinese medicine traceability system

**DOI:** 10.1186/s13020-022-00620-9

**Published:** 2022-05-28

**Authors:** Dazhong Zhao, Zunzhe Tian, Jing Cai, Juan He

**Affiliations:** School of Ecology and Environment, Northwestern Polytechnical University, Xi’an, 710072 China

**Keywords:** Chinese medicinal materials, TCM traceability system, Barcoded microbial spores

## Abstract

Traditional Chinese medicine (TCM) has a long history, rich clinical experience, and unique advantages in the prevention and treatment of diseases. The quality and safety of Chinese medicinal materials (CMMs) directly affect the clinical efficacy and development of the TCM industry. However, confused provenance, counterfeiting and adulteration of CMMs hinder the acceptance of its therapeutic benefits in modern society. Therefore, the establishment and improvement of a TCM traceability system would be conducive to the transparency of the CMMs production, distribution, and circulation, thereby improving drug safety and promoting industry development. This review discusses the challenges faced in the development of TCM traceability system, the technologies currently available for tracing CMMs, and the potential application of Barcoded Microbial Spores (BMS) to improve CMMs origin traceability and TCM traceability systems.

## Background

Traditional Chinese medicine (TCM) is a valuable asset accumulated by Chinese during their long-term struggle against diseases. Based on a unique system of knowledge and therapeutic procedures, TCM practitioners use a wide variety of Chinese medicinal materials (CMMs) from different plants and animals to prevent, diagnose, and treat diseases. The rapid growth in the use and demand for TCM in recent years has led to an increase in the demand for CMMs resources, with a consequent increase in the risk of quality degradation in the planting, processing, transporting, and storing stages. In addition, different environmental factors, such as topography, soil, climate, humidity, and light, can affect secondary metabolites, which are usually the bioactive components of CMMs [1]. Herbal processing can also affect the content, efficacy, and toxicity of the chemical components. Adulterated and counterfeit medicines also exist throughout the supply chain, from production to distribution [2]. Therefore, it is urgent to establish and improve the TCM traceability system to realize the transparency of information in all links of TCM circulation.

In the process of improving and enriching the TCM traceability system in China, some challenges we have to face and overcome. Firstly, the production of herbal medicine is still dominated by traditional agriculture, with the widely distributed planting sites, inexperienced growers, and region-specific CMMs names increasing the difficultly of traceability [3]. Secondly, the widely dispersed growing areas make it difficult to achieve large-scale transportation and efficient logistics. Furthermore, various woven bags, wire or rope, etc. such no unified packaging methods, make the CMMs lack of protection in the process of transportation, resulting in serious loss of quality of CMMs. Meanwhile, the imperfect supply chain and loopholes gives chances of adulteration and counterfeiting, which further increases the difficulty of tracing CMMs [4].

## Current TCM traceability technologies

TCM traceability systems based on Internet of Things (IoT) technology can record information on CMMs and ensure the stability of information flow. With the development of analytical instruments and the introduction of molecular biology technology, several traceability techniques have been developed, including chemical fingerprinting, DNA barcoding, and stable isotope tracing, which can sensitively and accurately identify the species, quality, and origin of CMMs [2, 4, 5]. Comparative analysis of the TCM traceability techniques is provided in Table 1.

**Table 1:** Technical parameters of traceability techniques

Technology type	Reliability	Repeatability	Information volume	Cost	Security
Fingerprint	Determine origin by identifying CMMs composition information, with average accuracy	Average	Contain correlation between fingerprints and origin	High	Only professionals or professional institutions can obtain information, with high security
DNA barcoding	Determine origin by identifying specific DNA sequences, with high accuracy	High	Contain correlation between gene and origin	High	Only professionals or professional institutions can obtain information, with high security
Isotope tracing	Determine origin based on characteristic elements, with average accuracy	High	Contain correlation between characteristic elements and origin	Gradual decline	Only professionals or professional institutions can obtain information, with high security
BMS	Determine origin by identifying DNA barcode sequences, with high accuracy	High	Contain production and distribution information	Gradual decline	Unencrypted and encrypted information can be added for both security and flexibility

Chemical fingerprinting can analyze CMMs through chromatographic or spectroscopic techniques to obtain corresponding chromatograms or spectrograms, which accurately identify the different chemical components in CMMs, referred to as TCM fingerprints [5]. Infrared spectroscopy, gas chromatography, and high-performance liquid chromatography are commonly used to characterize and authenticate the chemical components of CMMs [6]. The active compounds of the same CMMs can vary from one growing area to another due to differences in the growing environments, e.g., soil and humidity. Thus, fingerprint technology can determine planting areas based on fingerprint similarity and thus trace the origin of CMMs, although multiple batches of CMMs from different sources need to be analyzed in advance.

DNA barcoding enables standard and rapid identification of CMMs by selecting a relatively simple gene sequence with stable genetic variation across a species that can be easily amplified. The gene sequence identification method was first proposed in 2003 by Paul Hebert and his colleagues [7]. DNA barcoding can trace provenance based on the unique variant sequences of the same CMMs produced in different areas. This tracing technology does not require professional taxonomic knowledge and testing is fast, stable, and accurate, which is an important advancement in the appraisal of CMMs quality. Molecular identification of CMMs using DNA barcoding has seen rapid advancement and is now included in the Supplement of the Pharmacopoeia of the People’s Republic of China. Based on complete sample analysis, various DNA barcoding systems have been established to identify CMMs, such as the ITS2 core barcode and *psb*A-*trn*H complementary locus for identification of planta medica, and COI core barcode and ITS2 complementary locus for identification of animal medica [8]. However, certain processing methods can degrade DNA, thereby impacting identification.

Stable isotope tracing is based on differences in stable isotope compositions, which can be influenced by local environmental factors such as soil and moisture. Stable isotope technology can be applied to characterize the isotopes of CMMs from diverse geographical origins, and thus determine differences in different production areas to achieve accurate CMMs traceability and authenticity [9]. At present, stable isotopes used for origin traceability include hydrogen, carbon, oxygen and nitrogen. Strontium and lead are also gradually used to trace the origins of objects. Stable isotope tracing is not easily influenced by external factors, and CMMs origin analysis is accurate and efficient. However, the resolution of this method is not sufficient to distinguish CMMs, which are from nearby production areas.

Nanomolecular tags are difficult to counterfeit and can be applied to small and numerous objects, thus exhibiting good prospects in the field of TCM traceability. Porcupine is a molecular tagging system based on the presence or absence of synthetic DNA sequences. Using a portable nanopore device, the DNA-based tags can be read within seconds, showing considerable potential for the molecular tagging of different objects [10]. However, molecular markers applied to CMMs should have the characteristics of simple markers and be difficult to lose. A recent study by Springer's team from Harvard Medical School introduced a new microbial spore genetic marker technique, called Barcoded Microbial Spores (BMS). The BMS system can trace the origins of agricultural products, manufactured goods, as well as the CMMs, with the advantages such as persistence in the environment, scalability, rapid and facile decoding, and biocontainment [11].

## Application of BMS technology in CMMs traceability solutions

The BMS system identifies the provenance of objects within one hour at meter-scale resolution and near single-spore sensitivity and can be safely introduced into and recovered from the environment [11]. The system uses DNA-BMS mixtures to determine object provenance and the designed nonredundant DNA barcodes are integrated into the genomes of common microbial spores, which can be used to provide a nearly infinite set of identification codes. The spores have a tough structure that allows them to persist for long periods in harsh environments, thereby avoiding the risk of barcode loss during exposure. BMS can be manufactured at scale using standard cloning and culturing techniques and inoculated on the surface of plant stems and leaves by simple spraying. The cross-association of BMS-inoculated plants does not affect provenance determination. Barcodes can be identified by the SHERLOCK assay, a cas13a-based recombinant polymerase amplification (RPA) method, which allows rapid nucleic acid detection and its isothermal amplification properties are also suitable for field-deployable detection. The spores obtained by swabbing the plant surface are rapidly lysed by heat and sodium hydroxide methods, then tested using SHERLOCK and sequenced by Sanger to determine the origin of each leaf. The BMS approach uses nutrient-deficient strains of Bacillus subtilis and Saccharomyces cerevisiae that are commonly found in environmental and food samples, which require amino acid supplementation for growth. To ensure the synthetic spores do not grow naturally in the environment and cause adverse ecological effects, the BMS approach deletes the genes required for spore germination and growth or inactivates the spores by heating.

**Fig. 1:**
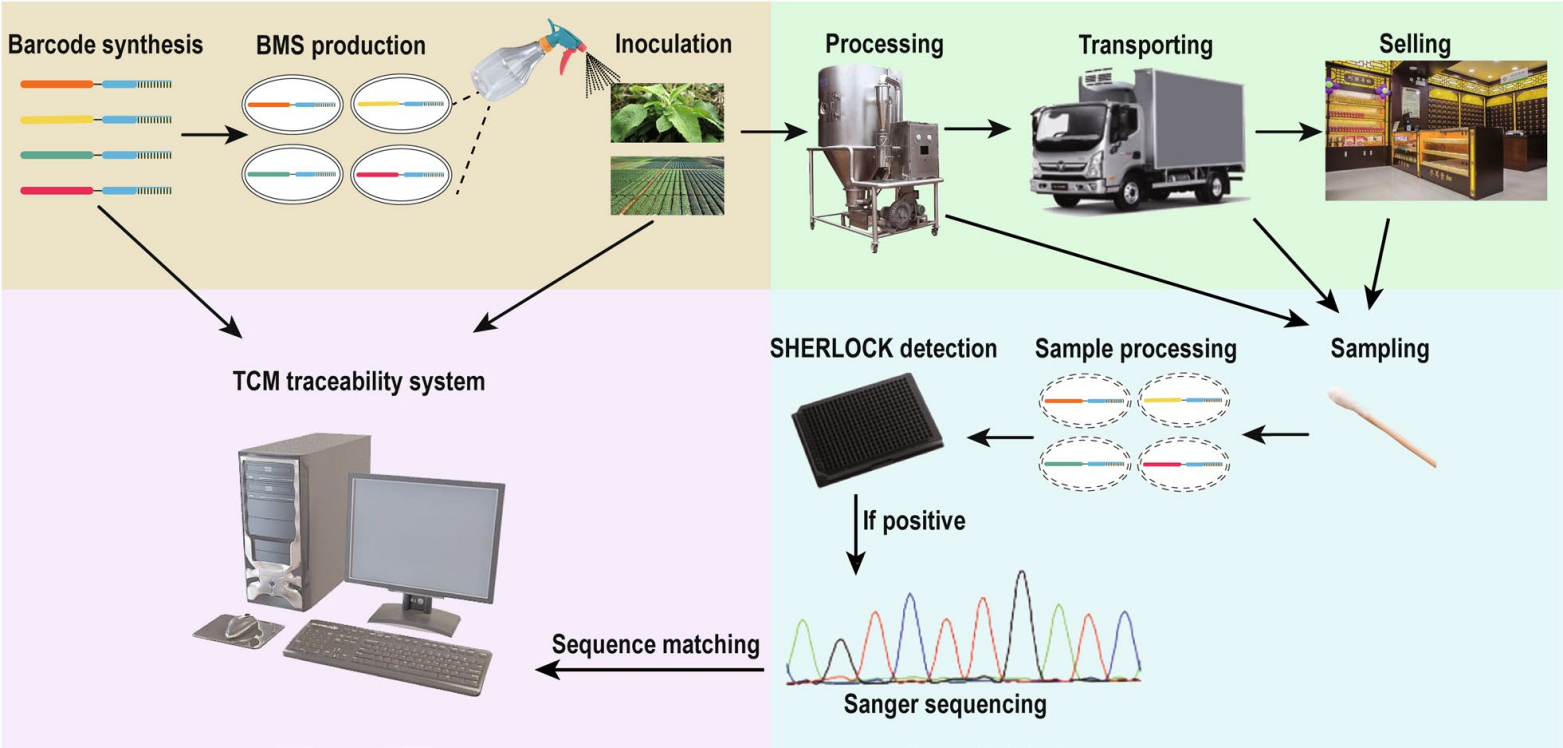
Schematic of entire BMS-based tracking procedure for traceability of Chinese medicinal materials

The BMS protocols for CMMs traceability are shown in Fig. 1. The first step is to design DNA sequences as tags. The first DNA sequence is selected as a shared group sequence of the same-species CMMs to facilitate high-throughput detection, while the second specific sequence is used to differentiate different provenances. The tandem DNA barcodes are integrated into the spores, which are then inoculated on the CMMs plant surface by spraying. Details on the DNA sequences in the BMS on all inoculated CMMs are recorded into the TCM traceability system to generate an origin and DNA sequence database as the basis for traceability. The BMS labels can be safely sprayed on food and remain detectable for several months [11]. At present, *Bacillus thuringiensis*, which is closely related to *Bacillus subtilis*, is widely used as a pesticide for many commercially available agricultural products [12]. The genomic DNA of *Bacillus thuringiensis* can be detected on store-bought products, even after washing, boiling, frying, and microwaving, indicating that the BMS tags will not be lost during CMMs processing. Detection of BMS only requires a swab to collect a sample from the CMMs surface. SHERLOCK analysis can then rapidly and sensitively detect the shared group sequence, thereby facilitating on-site testing for CMMs quality and identification during transport to ensure no adulteration or production of substandard medicines. After the CMMs arrive at their destination, Sanger sequencing is performed on the BMS-specific sequences, and the identified information is compared with the sequence information recorded in the database to determine the source of each CMM.

As an innovative microbial traceability technology, BMS, combined with genetic marker technology, distinguishes released microbial spores from those existing in the wild. BMS technology solves several common challenges that hinder the development of microbial source tracing technology and can improve TCM traceability. Synthetic spores can be mass-produced, thereby reducing the costs of BMS and improving applicability to a variety of CMMs. BMS technology simplifies the collection and verification of information from cultivation to sale, and can accelerate the promotion of TCM traceability nationwide, although validation across a wider range of real-world conditions is required. Furthermore, BMS can realize the transparency of information from provenance to market for both artificial cultivars with relatively concentrated production areas and wild CMMs with relatively dispersed distribution. The mature application of BMS technology shows great potential in standardizing the CMMs market and accelerating the international development of the TCM industry.

## Conclusions

The establishment of a TCM traceability system aims to achieve openness and transparency of information in the whole process of CMMs circulation, which would be conducive to the global development of TCM. BMS technology can accurately trace CMM provenance, and the use of shared group sequence and specific sequence in tandem provide a nearly infinite set of identification codes, which can meet the requirement that each CMM has its own barcode. The SHERLOCK assay, which can be performed on-site, and Sanger sequencing, which can identify specific sequences, make the entire process fast, convenient, and accurate. Thus, the use of BMS technology could significantly improve TCM traceability, guarantee the quality and safety of CMMs, and promote the sustainable and rapid growth of the TCM industry.

## Data Availability

Not applicable.

## Abbreviations

TCM: Traditional Chinese medicine
CMM: Chinese medicinal materials
BMS: Barcoded Microbial Spores
IoT: Internet of Things
RPA: Recombinant polymerase amplification

## References

[1] Sacks D, Baxter B, Campbell BCV, Carpenter JS, Cognard C, Dippel D (2018). Multisociety consensus quality improvement revised consensus statement for endovascular therapy of acute ischemic stroke. Int J Stroke.

[2] Xu M, Huang B, Gao F, Zhai C, Yang Y, Li L (2019). Assesment of adulterated traditional Chinese medicines in China: 2003–2017. Front Pharmacol.

[3] Du XL, Shi MY (2019). Application of DNA barcode technology in traceability of traditional Chinese medicine. Comput Knowl Technol.

[4] Yang HX, Sun LJ (2017). Protection and development of Chinese materia medica through modern logistics system. Chin J Inform Trad Chin Med.

[5] Liu X, Jiang W, Su M, Sun Y, Liu H, Nie L (2020). Quality evaluation of traditional Chinese medicines based on fingerprinting. J Sep Sci.

[6] Wang L, Wang Q, Wang Y, Wang Y (2021). Comparison of geographical traceability of wild and cultivated *Macrohyporia cocos* with different data fusion approaches. J Anal Methods Chem.

[7] Hebert PDN, Cywinska A, Ball SL, DeWaard JR (2003). Biological identifications through DNA barcodes. Proc R Soc Lond B.

[8] Taraporevala S, Sahin M, Yorek N, Torres JP, Mendes EG, Toenders FGC (2013). Principles for molecular identification of traditional Chinese materia medica using DNA barcoding. China J Chin Mater Med.

[9] Yu DX, Guo S, Yang J, Yan H, Zhang ZY, Duan JA (2022). Application and prospect of stable isotope technology in tracing geographical origin of Chinese herbal medicines. China J Chin Mater Med.

[10] Doroschak K, Zhang K, Queen M, Mandyam A, Strauss K, Ceze L (2020). Rapid and robust assembly and decoding of molecular tags with DNA-based nanopore signatures. Nat Commun.

[11] Qian J, Lu ZX, Mancuso CP, Jhuang HY, Barajas-ornelas RC, Boswell SA (2020). Barcoded microbial system for high-resolution object provenance. Science.

[12] Sanahuja G, Banakar R, Twyman RM, Capell T, Christou P (2011). *Bacillus thuringiensis*: a century of research, development and commercial applications. Plant Biotechnol J.

